# Supplementary material for: Rapid turnover of life-cycle-related genes in the brown algae
Source: Genome Biol. 2019 Feb 14;20:35. doi: 10.1186/s13059-019-1630-6 (PMC6374913; doi:10.1186/s13059-019-1630-6)
Supplement: Supplementary file 3 — Protein alignments, corresponding Gblocks html file and CDS sequences for the lineage conserved gametophyte-biased genes. (ZIP 2109 kb) [file 13059_2019_1630_MOESM3_ESM.zip › Con_GA_alignments/SJ03836.aa_ali.fasta-gb.htm]

SJ03836.aa.fasta.trans.aa\_ali.fasta


## Gblocks 0.91b Results

Processed file: **SJ03836.aa.fasta.trans.aa\_ali.fasta**  
Number of sequences: **2**  
Alignment assumed to be: **Protein**  
New number of positions: **234** (selected positions are underlined in blue)

```
                         10        20        30        40        50        60
                 =========+=========+=========+=========+=========+=========+
SJ03836          MGARRGDGESSEGWQDVGHGDGSAVPCSRRSMLRSLGLTGAAVVCGQALIGQSGPAMAQD
Mac.55817.1      MVSSR------------------PKLAPR---IVQLGLTGAAVVCGQALIGKSGPAIAQD
                                                    #########################


                         70        80        90       100       110       120
                 =========+=========+=========+=========+=========+=========+
SJ03836          ELPTVGKITKTGVKYFDYRVGEGSTPRWGQDCIVRFVMYGRSSPDEKLTKIHSSDDNKET
Mac.55817.1      ELPTVGKITKTGVKYFDYRVGEGSTPRWGQDCIVRFVMYGRSSPDEKLTKIHSSDDNKET
                 ############################################################


                        130       140       150       160       170       180
                 =========+=========+=========+=========+=========+=========+
SJ03836          YVFKHGNGLQIKGMEEGIHSMRVGGKRRVIMPQQLGYVVQGLGPYPADPRKRDVLVKVLT
Mac.55817.1      YVFKHGNGLQIKGMEEGIHSMRVGGKRRVIMPQQLGYAVQGLGPYPADPRKRDVLVKVLT
                 ############################################################


                        190       200       210       220       230       240
                 =========+=========+=========+=========+=========+=========+
SJ03836          AFEGSKKGELVMDVELLDAFDDEADQGYYEDETVSVEELQDIVDQTGRFSGGMPFEKLPG
Mac.55817.1      AFEGTKKGELVMDVDLLDAFDDEADQGYYEDETVSVEELQDIVDQTGRFSGGMPFEKLPG
                 ############################################################


                        250       260       270
                 =========+=========+=========+
SJ03836          QVASPLDDGVSSSPRGNKLPGQLGQDLGGE
Mac.55817.1      QVASPLDDGVASPSRGNKLPGQLGQDIGGT
                 #############################
```

```
Parameters used
Minimum Number Of Sequences For A Conserved Position: 2
Minimum Number Of Sequences For A Flanking Position: 2
Maximum Number Of Contiguous Nonconserved Positions: 8
Minimum Length Of A Block: 10
Allowed Gap Positions: None
Use Similarity Matrices: Yes
```

```
Flank positions of the 1 selected block(s)
Flanks: [36  269]  

New number of positions in SJ03836.aa.fasta.trans.aa_ali.fasta-gb:  234  (86% of the original 270 positions)
```
